# Stress-delta B-type Natriuretic Peptide Levels as a Test for Inducible Myocardial Ischemia: A Systematic Review and Meta-Analysis

**DOI:** 10.7759/cureus.7165

**Published:** 2020-03-02

**Authors:** Sopagna Kheang, Clarissa G Rodrigues, Joao Ricardo N Vissoci, Almujtaba Hassan, Christian Muller, Deborah Muller, Alexander T Limkakeng

**Affiliations:** 1 Emergency Medicine, Duke University School of Medicine, Durham, USA; 2 Board of Directors, Global Research and Innovation Network, Joinville, BRA; 3 Instituto De Cardiologia Do Rs, Fundação Universitária De Cardiologia, Porto Alegre, BRA; 4 Emergency Medicine, Duke Global Health Institute, Duke University School of Medicine, Durham, USA; 5 Cardiovascular Research Institute, University Hospital of Basel, Basel, CHE

**Keywords:** delta bnp, delta nt-pro-bnp, biomarkers, acute coronary syndrome, inducible myocardial ischemia, n-terminal pro-b-type natriuretic peptide (nt-pro-bnp), b-type natriuretic peptide (bnp)

## Abstract

Background

Cardiac ischemia induces myocardial dysfunction and ventricular wall stretch, which causes the release of B-type natriuretic peptide (BNP) into the bloodstream. However, it is unclear whether inducible ischemia produces a significant change in BNP levels (“stress delta-BNP”). The objective of this study was to determine the utility of stress-delta BNP levels and its precursor NT-proBNP for detecting inducible myocardial ischemia during cardiac stress testing.

Methods

We conducted a systematic review and meta-analysis. We searched PubMed, EMBASE, Web of Science, Cumulative Index of Nursing and Allied Health Literature (CINAHL), and Ovid. Studies examining the changes in levels of BNP and its precursor, N-terminal pro-B-type natriuretic peptide (NT-proBNP), after an exercise cardiac stress test were included. Two reviewers independently analyzed titles and abstracts. Abstracts that did not provide enough information regarding eligibility criteria were kept for full-text evaluation. The same two reviewers also performed data extraction for analyses. Any disagreement was resolved by a consensus and, if it persisted, by a third reviewer adjudication. We report the median and mean values in studies in the order of sample size.

Results

A total of 15 studies met the inclusion criteria. Nine studies reported results in medians and six studies reported results in means. Of the nine studies, five assessed BNP alone, three assessed NT-proBNP, and one assessed both. Due to the non-normal distribution of results in these studies, they could not be meta-analyzed. Of the six studies that reported results in means, three assessed BNP and three assessed NT-proBNP. The standardized difference between normal and ischemic patients' stress-delta BNP values was -0.39 (95% confidence interval (CI): -0.61; -0.17) in a fixed-effects model and -0.73 (95% CI: -1.72; 0.28) in the random-effects model with high heterogeneity (I^2 = 94%, Q test P = 0.001). For NT-proBNP, the meta-analysis model showed no significant difference between the stress-delta test for ischemic and normal patients (standardized mean difference (SMD): -0.02, 95% CI: -0.31; 0.28). Patients without inducible ischemia appeared to have a lower baseline BNP and NT-proBNP compared to patients with inducible ischemia by stress testing. Although some studies report higher stress-delta BNP in the ischemic group, this pattern was not seen consistently across studies. There was high heterogeneity across studies which was not robust to sensitivity analysis. A random-effects model failed to find statistically significant differences in stress-delta BNP or NT-proBNP.

Conclusions

We failed to find a relationship between stress-delta BNP or NT-proBNP and the presence or absence of ischemia. This may be due to high heterogeneity in the underlying studies.

## Introduction

Acute coronary syndrome (ACS) remains a deadly and costly condition. One of the most common ways it is assessed is through a cardiac stress test. In this test, the patient’s heart is stressed using either exercise or medications to increase its oxygen demand. The patient’s heart is then assessed for inducible myocardial ischemia (a precursor to ACS) using some form of diagnostic imaging [1-3]. Accordingly, cardiac stress tests require sophisticated equipment and trained personnel to perform and interpret. This limits the test’s availability and affordability, leading to frequent hospitalization for patients with possible ACS. A different paradigm is needed to diagnose ACS.

One potential solution would be to utilize bloodstream biomarkers in the place of imaging studies to detect inducible myocardial ischemia. Multiple studies have attempted to determine whether there are dynamic biomarker correlates of inducible myocardial ischemia. B-type natriuretic peptide (BNP) is released by the myocardium in response to ventricular wall stretch. It has been hypothesized that BNP would also be released in myocardial ischemia, given that induced myocardial ischemia is associated with abnormal ventricular wall motion in the affected area. Prior individual studies examining changes in BNP as a predictor of stress test-induced myocardial ischemia were underpowered to make definitive conclusions and have findings that contradict one another [4-12]. Others have also studied the BNP precursor, N-terminal pro-B-type natriuretic peptide (NT-proBNP), which has similar characteristics as a biomarker and similarly found contradictory results [10, 13-15].

If clinicians could reliably use changes in bloodstream biomarkers following stress to detect myocardial ischemia, the need for highly trained personnel and sophisticated equipment for an imaging study could be precluded. Using “stress-delta” biomarkers in such a manner could have multiple advantages, including increased cost-effectiveness, efficiency, availability, and reproducibility. The goal of this systematic review and meta-analysis is to determine the utility of stress-delta BNP levels and its precursor, NT-proBNP, for detecting inducible myocardial ischemia during cardiac stress testing.

## Materials and methods

This systematic review is reported in accordance with the Preferred Reporting Items for Systematic Review and Meta-analyses (PRISMA) statement [16].

Eligibility criteria

Studies were eligible if they examined the changes in BNP or its precursor, NT-proBNP, before and after a stress test to assess myocardial ischemia in patients with known or suspected to have coronary artery disease. We excluded studies that examined these biomarkers in patients with systolic heart failure and in studies using them as prognostic rather than diagnostic markers. We also excluded studies that failed to exclude patients with congestive heart failure and studies that used pharmacological stress testing.

Information sources

We searched the following electronic databases: PubMed, Excerpta Medica database (EMBASE), Web of Science, Cumulative Index of Nursing and Allied Health Literature (CINAHL), and Ovid. In addition, we searched the references of the included articles manually for further studies that would meet the eligibility criteria. When necessary, we also contacted authors of eligible studies for data not reported.

Search

The initial search comprised the Medical Subject Headings (MeSH) terms “myocardial ischemia,” “brain natriuretic peptide,” “BNP,” “diagnostic test,” and related entry terms. The complete search strategy used for the PubMed and EMBASE databases is shown in Table 1. We did not use limits for language and date when conducting the searches.

**Table 1:** PubMed Search Strategy

Search	Query
#4	Search (#1 AND #2 AND #3)
#3	Search ("Natriuretic Peptide, Brain"[Mesh] OR "Peptide, Brain Natriuretic"[All Fields] OR "Brain Natriuretic Peptide"[All Fields] OR "BNP-32"[All Fields] OR "BNP 32"[All Fields] OR "Nesiritide"[All Fields] OR "B-Type Natriuretic Peptide"[All Fields] OR "Natriuretic Peptide, OR B-Type"[All Fields] OR "BNP Gene Product"[All Fields] OR "Type-B Natriuretic Peptide"[All Fields] OR "Natriuretic Peptide, Type-B"[All Fields] OR "Type B Natriuretic Peptide"[All Fields] OR "Natriuretic Peptide Type-B"[All Fields] OR "Natriuretic Peptide Type B"[All Fields] OR "Natriuretic Factor-32"[All Fields] OR "Natriuretic Factor 32"[All Fields] OR "Brain Natriuretic Peptide-32"[All Fields] OR "Brain Natriuretic Peptide 32"[All Fields] OR "Natriuretic Peptide-32, Brain"[All Fields] OR "Peptide-32, Brain Natriuretic"[All Fields] OR "Ventricular Natriuretic Peptide, B-type"[All Fields] OR "Ventricular Natriuretic Peptide, B type"[All Fields] OR "Natrecor"[All Fields] OR "Basic natriuretic peptide"[All Fields] OR "B-type Natriuretic Peptide (BNP) blood test"[All Fields] OR "B-type Natriuretic Peptide blood test"[All Fields] OR "B-type Natriuretic Peptide (BNP)"[All Fields] OR "BNP"[All Fields] OR "BNP blood test"[All Fields])
#2	Search ("Diagnostic Test Accuracy"[All Fields] OR "Diagnostic Study"[All Fields] OR "Sensitivity and Specificity"[Mesh] OR "Specificity and Sensitivity"[All Fields] OR "Sensitivity"[All Fields] OR "Specificity"[All Fields])
#1	Search ("Myocardial Ischemia"[Mesh] OR "Ischemia, Myocardial"[All Fields] OR "Ischemias, Myocardial"[All Fields] OR "Myocardial Ischemias"[All Fields] OR "Ischemic Heart Disease"[All Fields] OR "Heart Disease, Ischemic"[All Fields] OR "Disease, Ischemic Heart"[All Fields] OR "Diseases, Ischemic Heart"[All Fields] OR "Heart Diseases, Ischemic"[All Fields] OR "Ischemic Heart Diseases"[All Fields] OR "Coronary Artery Disease"[Mesh] OR "Artery Disease, Coronary"[All Fields] OR "Artery Diseases, Coronary"[All Fields] OR "Coronary Artery Diseases"[All Fields] OR "Disease, Coronary Artery"[All Fields] OR "Diseases, Coronary Artery"[All Fields] OR "Coronary Arteriosclerosis"[All Fields] OR "Arterioscleroses, Coronary"[All Fields] OR "Coronary Arterioscleroses"[All Fields] OR "Atherosclerosis, Coronary"[All Fields] OR "Atheroscleroses, Coronary"[All Fields] OR "Coronary Atheroscleroses"[All Fields] OR "Coronary Atherosclerosis"[All Fields] OR "Arteriosclerosis, Coronary"[All Fields])

Study selection

Two reviewers (SK and AH) independently evaluated titles and abstracts of the retrieved articles. Abstracts that did not provide enough information regarding the eligibility criteria were kept for full-text evaluation. Reviewers independently evaluated full-text articles and determined study eligibility. Disagreements were resolved by consensus, and if any disagreement persisted, a third reviewer’s opinion (ATL) was sought.

Risk of bias

The risk of bias was evaluated by ranking each study according to standard factors using the Quality Assessment of Diagnostic Accuracy Studies (QUADAS) tool. Bias assessment considered each study’s representativeness, reference standard acceptability, the delay between the index and reference standard, avoidance of partial or differential verification, avoidance of incorporation of index test into reference standard, blinding, the relevance of results, reporting of intermediate/indeterminate results, and explanation of withdrawals.

Data extraction

Two reviewers (SK and AH) independently conducted the data extraction and disagreements were resolved by the third reviewer (ATL). General characteristics of the studies were collected, such as study design, study settings, population description, age, and data collection time range. Most importantly, the required data for this study, including baseline and ending BNP or NT-Pro-BNP, disease prevalence, type of stress, and times that outcome was measured, were also collected.

Data analysis

Due to a wide skew in BNP and NT-Pro-BNP distributions in the studies that met the eligibility criteria, as well as the wide variation in methods among the studies, a meta-analysis was only performed on the studies reporting mean and a variability measure for both outcomes. Thus, to summarize all studies, we also qualitatively report studies’ median and mean values ordered by sample sizes.

Meta-analysis models were built for BNP and NT-Pro-BNP delta values (difference) between pre- and post-stress testing comparing normal and ischemic patients. Heterogeneity across the studies was examined through the Cochrane's Q (considering p-values lower than 0.1 to indicators of heterogeneity), H and I2statistics [17]. High I2 values indicate high heterogeneity with a proposed categorization of 25% (low), 45% (moderate), and 75% (high) [17]. In our study, we adopted the interpretation of the fixed-effect model because our main goal is to obtain the true-effect of BNP and NT-Pro-BNP along the different population sizes reported. Sensitivity analyses were conducted by including different subsets of articles based on their quality rank, which allowed us to test the robustness of our results. A forest plot was constructed to depict these results. All analyses were performed through the R language software (R-Project, version 3.1.0, 2016), specifically through the meta package and metafor package [18].

## Results

Study selection

**Figure 1:**
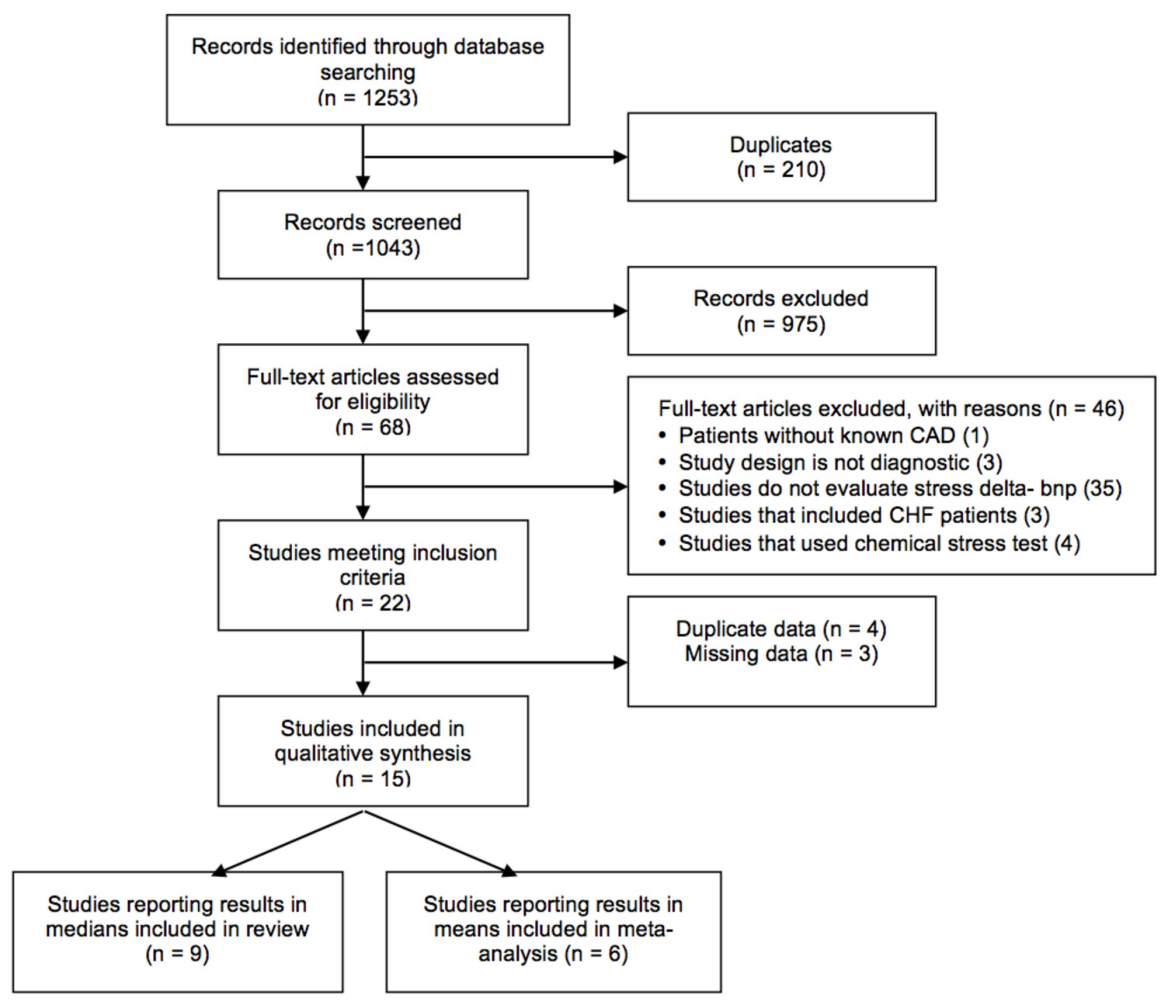
Study selection flowchart BNP: B-type natriuretic peptide; CAD: coronary artery disease; CHF: congestive heart failure

A total of 1,253 records were identified after searching through the various databases. Sixty-eight full-text articles were assessed for eligibility and 46 were excluded, leaving 22 articles that met the inclusion criteria. After further evaluation, only 15 studies contained unique data and were included in our synthesis. Of the 15 studies, six studies reported results in means and nine studies reported results in medians and were included in this systematic review (Figure 1).

Study characteristics

The population characteristics of these studies can be seen in Table 2. The mean age of participants ranged from 57 to 66 years old. The prevalence of ischemia in the studies ranged between 16.7% and 78%. The number of patients in each study ranged between 44 and 274. Only studies with exercise stress testing were included.

**Table 2:** Characteristics of Included Studies *studies examining both BNP and NT-proBNP BNP: B-type natriuretic peptide; CAD: coronary artery disease; MPI: myocardial perfusion imaging; NT-proBNP: N-terminal pro-B-type natriuretic peptide; SPECT: single-photon emission computed tomography

Study	Year	Setting	Study Population	Mean Age	Men (%)	Type of Stress	N	Prevalence of ischemia
BNP								
Lee et al. [7]	2014	University Hospital of Basel	Consecutive patients with suspected CAD referred for stress testing	62	68%	Bicycle exercise with SPECT	274	37.6%
Staub et al. [8]	2006	University Hospital of Basel	Consecutive patients referred for CAD evaluation by bicycle ergometry MPI SPECT	63	70%	Bicycle exercise MPI SPECT	256	49.6%
Zaid et al. [4]	2006	Bnai-Zion Medical Center	Consecutive patients referred for chest pain evaluation	59	67%	Exercise with nuclear perfusion imaging	203	52%
Moller et al. [9]	2008	Mayo Clinic, Rochester	Patients referred for evaluation of angina or exertional dyspnea	65	55%	Bicycle exercise echo	140	46%
Paraskevaidis et al. [5]	2011	Outpatient clinic	Consecutive patients with chest pain	58.7	80%	Treadmill exercise	100	78%
*Foote et al. [10]	2004	Dartmouth Hitchcock Medical Center	Consecutive patients with CAD referred for MPI SPECT	58.8	84%	Exercise with MPI SPECT	74	54%
Marumoto et al. [6]	1995	-	35 patients with angiographically proven angina and 35 angiographically normal patients	61.1	67%	Exercise stress test with SPECT	70	50%
Bergeron et al. [11]	2006	-	Patients referred for clinically indicated exercise echo	66	82%	Treadmill exercise with echo	60	31.7%
Win et al. [12]	2005	-	Patients undergoing treadmill exercise for evaluation of chest pain or screening for ischemia	57.2	68%	Treadmill exercise with SPECT	60	16.7%
NT-Pro BNP								
Staub et al. [13]	2005	University Hospital Basel	Consecutive patients referred for CAD evaluation	63	70%	Bicycle exercise with SPECT	260	49.6%
Vanzetto et al. [14]	2007	University Hospital of Grenoble	Patients with known stable CAD referred for exercise MPI	61	88%	Bicycle exercise MPI	102	55.9%
Van der Zee et al. [15]	2009	-	Consecutive patients referred for evaluation of inducible ischemia	61	64%	Bicycle exercise with SPECT	101	36.6%
Başkurt et al. [19]	2011	Outpatient clinic	Patients with a history of exercise-induced angina or atypical angina	57	63%	Exercise stress test	96	50%
*Foote et al. [12]	2004	Dartmouth Hitchcock Medical Center	Consecutive patients with CAD referred for MPI SPECT	58.8	84%	Exercise with MPI SPECT	74	54%
Chatha et al. [20]	2006	Rapid access chest pain clinic	All patients with chest pain from rapid access chest pain clinic	58	46%	Exercise stress test	59	23.7%
Zhu et al. [21]	2010	Zhongshan Hospital	Patients with chest pain and suspected CAD	-	-	Treadmill exercise	44	56.8%

Out of the total 15 studies included, eight studies examined BNP, six examined NT-proBNP, and one examined both. Table 3 depicts the data in order of sample size for studies examining BNP with median and mean stress-deltas, respectively. Eight of the nine BNP studies demonstrated a higher stress-delta value in patients with ischemic stress tests than in patients with normal stress tests. These studies represent 1,177 of the 1,237 patients (95%) with stress-delta BNP.

**Table 3:** Stress-delta BNP/NT-proBNP Studies (ng/L) “Delta-delta” refers to the stress-delta BNP for patients with ischemia on stress imaging, minus the stress-delta BNP for patients with normal stress imaging. BNP: B-type natriuretic peptide; IQR: interquartile range; N: number; NT-proBNP: N-terminal pro-B-type natriuretic peptide; SD: standard deviation

Authors	Ischemic Patients					Normal Patients				Delta-Delta	Total N	
	N	Baseline	Post	Delta		N	Baseline	Post	Delta		
Median BNP (IQR)											
Lee et al. [7]	103	105.7 (57.1 - 176.9)	130.5 (74.3 - 260.9)	24.8		171	56.6 (30.4 - 94.3)	72.4 (44.4 - 116.3)	15.8	9	274
Staub et al. [8]	127	70.8 (34.8 - 146.5)	87.5 (42.2 - 173.6)	16.7		129	38.1 (19.6 - 81.4)	52.2 (24.7 - 102.3)	14.1	2.6	256
Moller et al. [9]	65	19 (8.8 - 34.6)	29.6 (17.6 - 46.5)	10.6		75	12.1 (4.6 - 26.9)	16.3 (7.1 - 34.1)	4.2	6.4	140
Foote et al. [10]	40	40.5 (24 - 54)	77 -	36.5 (15 - 49.5)		34	16.5 (9.5 - 30.5)	24 -	7.5 (3.5 - 17.5)	29	74
Win et al. [12]	10	13.4 (9.5 - 30.6)	26.7 (19.3 - 61.5)	13.2		50	15.05 (7 - 37.7)	34.7 (14.9 - 67.6)	19.65	-6.45	60
Bergeron et al. [11]	19	24 (10 - 69)	50 (30 - 94)	26		41	15 (8.1 - 24)	29 (13 - 40)	14	12	60
Mean BNP (SD)											
Zaid et al. [4]	106	117 (+/- 292)	149 (+/- 356)	32		97	50 (+/- 66)	67 (+/- 88)	17	15	203
Paraskevaidis et al. [5]	78	21.8 (+/- 15.3)	69.9 (+/- 63.2)	48.1		22	14.2 (+/- 17.0)	38.2 (+/- 51.1)	24	24.1	100
Marumoto et al. [6]	35	2.8 (+/- 0.8)	6.9 (+/- 2.6)	4.1		35	2.7 (+/- 0.7)	2.9 (+/- 1.0)	0.2	3.9	70
Median Pro-BNP (IQR)											
Staub et al. [13]	129	155 (84 - 360)	169 (90 - 391)	14		131	91 (43 - 228)	100 (50 - 244)	9	5	260
Vanzetto et al. [14]	57	182 (97 - 265)	212 (104 - 315)	30		45	85 (44 - 164)	99 (50 - 179)	14	16	102
Van der Zee et al. [15]	37	184 (57 - 386)	214 -	30 (7 - 45)		64	74 (21 - 255)	89 -	15 (4 - 46)	15	101
Foote et al. [10]	40	120.5 (76 - 158)	135 -	14.5 (10.5 - 19.5)		34	53.5 (28 - 74)	57.5 -	4 (0.5 - 9.5)	10.5	74
Mean Pro-BNP (SD)											
Başkurt et al. [19]	48	175.1 (+/- 392.3)	201.5 (+/- 461.6)	26.4		48	92.2 (+/- 130.5)	102.5 (+/- 139.2)	10.3	16.1	96
Chatha et al. [20]	14	71.4 (+/- 41.2)	76.8 (+/- 44.0)	5.4		45	54 (+/- 61.2)	60.1 (+/- 69.0)	6.1	-0.7	59
Zhu et al. [21]	25	187.97 (+/- 166.29)	197 (+/- 178.82)	9.03		19	107.27 (+/- 105.15)	117.911 (+/- 93.34)	10.64	-1.61	44

Table 3 also demonstrates the data from studies examining NT-proBNP with median and mean stress-deltas, respectively. Five of the seven studies demonstrated that patients with ischemic stress tests had higher stress-delta values compared to normal patients. These studies represent 633 of the 736 patients (86%) with stress-delta NT-proBNP calculated.

Van der Zee et al. [15] assessed the change of NT-proBNP at time-points greater than two hours post-stress. The study appears to show similar results at the time-points greater than two hours. In fact, this study shows higher stress-delta values at time-points greater than two hours compared to immediately post-stress.

Risk of bias

**Figure 2:**
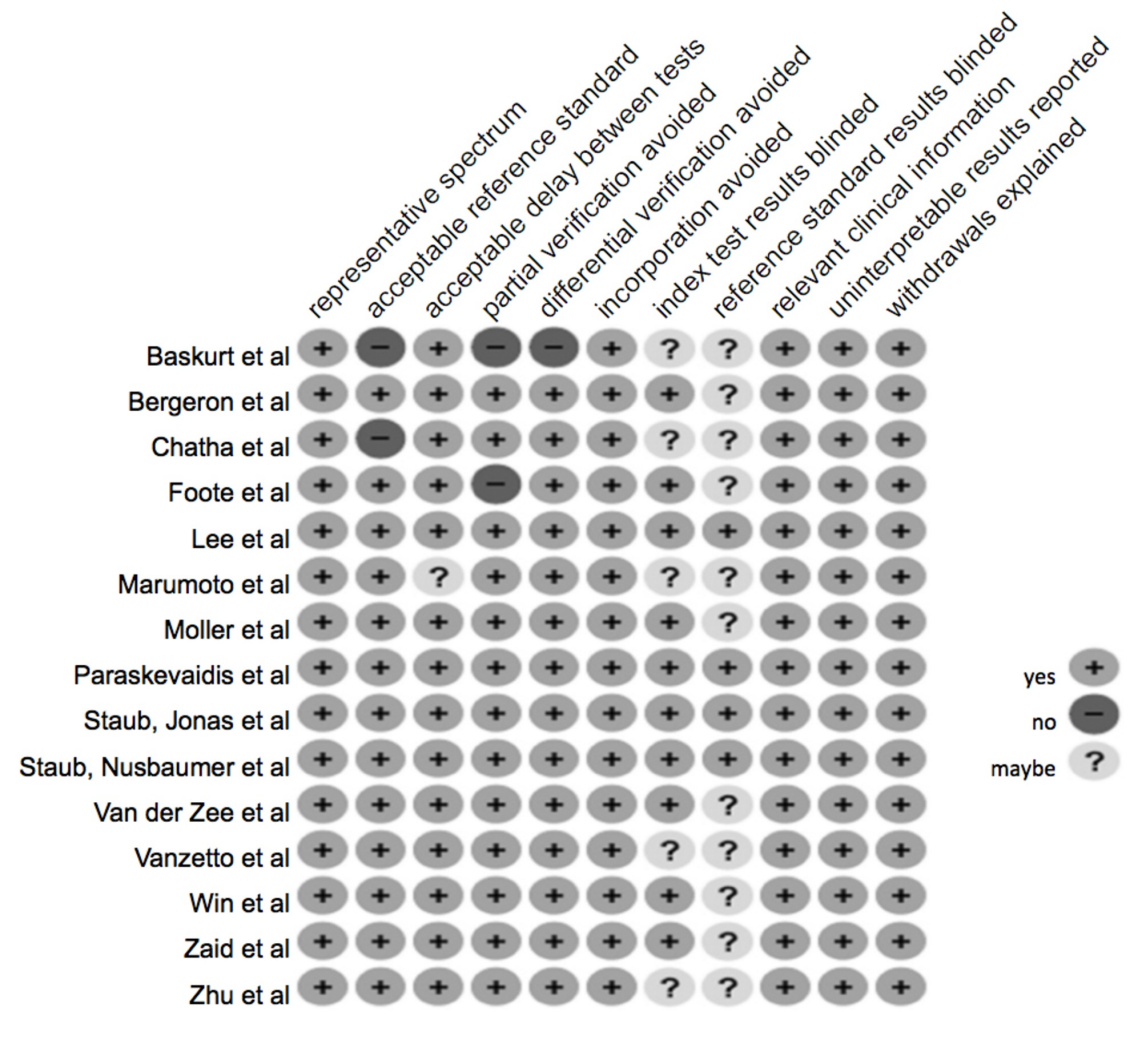
Assessment of risk [19, 11, 20, 10, 7, 6, 9, 5, 13, 8, 15, 14, 12, 4, 21]

Our bias assessment results are demonstrated in Figure 2. Four studies (Paraskevaidis et al., Lee et al., Staub et al., and Staub et al.) addressed all of the 11 characteristics evaluated [5, 7-8, 13]. The remaining 11 studies did not address whether the reference standard results were blinded and five of those studies also did not mention if the index test results were blinded. Studies by Başkurt et al. and Chatha et al. used an exercise treadmill test alone without imaging as a reference standard for the evaluation of coronary artery disease [19-20]. Foote et al. and Başkurt et al. were also at risk for partial verification [10, 19]. Başkurt et al. only evaluated with angiography if the exercise stress test was positive [19]. Foote et al. only used an exercise treadmill in healthy volunteers while using an exercise treadmill, as well as imaging, in the population with suspected coronary artery disease [10].

Meta-analysis

**Figure 3:**
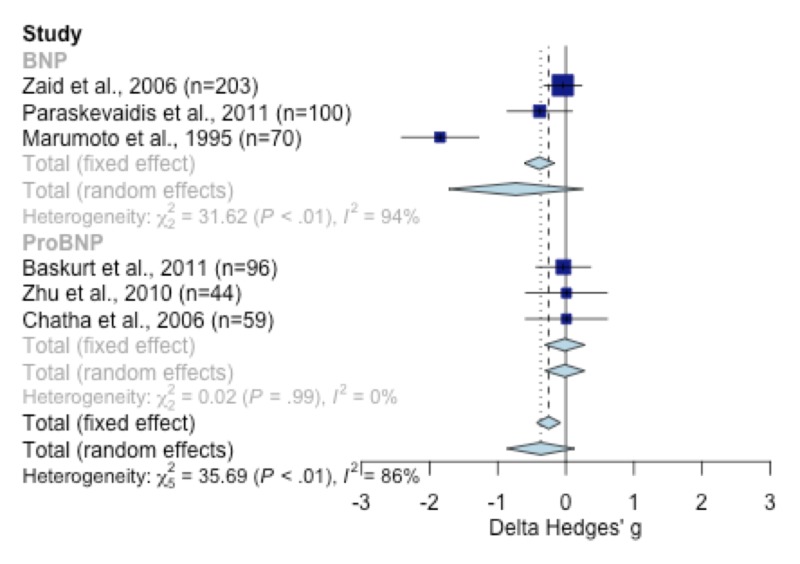
Meta-analysis of studies reporting mean values BNP [4-6]; ProBNP [19, 21, 20] BNP: B-type natriuretic peptide; ProBNP: pro-B-type natriuretic peptide

The standardized difference between normal and ischemic patients’ stress-delta BNP values was -0.39 (95% confidence interval (CI): -0.61; -0.17) in a fixed-effect model and -0.73 (95% CI: -1.72; 0.28) in the random-effects model (Figure 3). The model revealed high heterogeneity (I2 = 94%, Q test P = 0.001). Sensitivity analysis showed that excluding the study by Marumoto et al. [6] would produce a significant improvement to the model, reaching almost no heterogeneity (I2 = 0%, Q test P = 0.001), and a decrease in the estimated pooled standardized mean difference to -0.13 (CI 95%: -0.37; 0.11). Overall, the results correspond to a homogeneous model and show that ischemic patients had a slightly higher BNP delta than normal patients, although the difference was not statistically significant.

For NT-proBNP, the meta-analysis model showed no significant difference between the stress-delta test for ischemic and normal patients (standardized mean difference (SMD): -0.02, 95% confidence interval (CI): -0.31, 0.28). The NT-proBNP delta values for ischemic patients were similar to the normal patient group in a low heterogeneity fixed-effects model (Figure 11).

## Discussion

While some have questioned the utility of routine stress testing, it remains the guideline-recommended care for patients presenting with symptoms of the potential acute coronary syndrome [22-24]. Routine stress testing has further come into question in light of findings that coronary interventions in stable patients with positive stress tests do not prevent future acute myocardial infarction or death [25]. The ability to utilize a bloodstream biomarker to reliably detect inducible myocardial ischemia could reduce the need for sophisticated equipment and highly trained personnel currently used for stress testing [1-3]. Since the imaging component of contemporary stress testing is not usually available 24 hours per day, the need for stress testing drives many of the hospitalizations for patients with symptoms of acute coronary syndrome. A biomarker-based stress test, therefore, could make ACS assessment more affordable and efficient since laboratory testing is routinely available continuously in most hospitals.

It has long been recognized that BNP is released by the myocardium in response to ventricular wall stretch [26]. This has been observed in patients with chronic and acute-on-chronic congestive heart failure [27]. It has, therefore, been hypothesized that BNP would also be released in the setting of myocardial ischemia, given the observation that induced myocardial ischemia is associated with abnormal ventricular wall motion activity [26, 28]. A prior meta-analysis examined whether a single, resting BNP, or NT-proBNP level can predict inducible myocardial ischemia on stress testing and found relatively high diagnostic test characteristics [29].

Accordingly, we attempted to systematically review and synthesize the existing literature exploring the relationship of dynamic BNP release from induced myocardial ischemia occurring during stress testing by examining the stress-delta BNP levels. In our analysis, patients without inducible ischemia appeared to have lower baseline BNP and NT-proBNP values compared to patients with inducible ischemia by stress testing. Stress-delta BNP values were slightly higher in patients with inducible ischemia. However, we were unable to definitively identify a relationship between inducible ischemia and statistically different stress-delta BNP or NT-proBNP values after sensitivity analysis.

This systematic review and analysis have several strengths. We conducted a comprehensive search of various databases in addition to going through all citations of the included studies. We used standardized definitions of inclusion and exclusion criteria and trained abstractors with excellent agreement.

Qualitative analysis of the results suggests that there is not a strong relationship between median stress-delta BNP and inducible ischemia when the blood samples are drawn at peak exercise or immediately after stress testing. Thus, the timing of blood sampling may play a critical role in assessing the utility of any stress-delta biomarker paradigm.

We note some limitations to our systematic review. The study population of included papers appears to have a wide range of pretest probability of disease among subjects, and there was a wide range of prevalence of ischemia noted as well. There was a great deal of heterogeneity in the studies examined with regard to methods and results. There are many factors that can influence BNP levels, and although we eliminated studies that included patients with systolic heart failure, other causes of elevated baseline BNP that could skew results (such as valvular disorders, pulmonary hypertension, and left ventricular diastolic dysfunction) were not consistently controlled. There was variation in the BNP and NT-proBNP assays used. There was also no standardization of blood draw time points; some studies sampled during peak exercise while others were drawn within minutes after finishing. Further increase after stress may occur and thus requires further investigation. NT-proBNP has a longer half-life of two to three hours and has a higher baseline compared to BNP. Therefore, small absolute increases may be harder to detect [30]. The study by Van Der Zee et al. examining changes in NT-proBNP over time after stress showed a peak change immediately at peak exercise, at the second peak at four hours in patients with ischemia, and at five hours in patients without ischemia [15]. In addition, treadmill exercise might not be a significant enough stressor to induce myocardial ischemia or it might produce an ischemic state that is too short to create significant stress-delta changes. Finally, stress modalities for inducing myocardial ischemia may also affect the result. The pharmacologic stress test, such as dobutamine and dipyridamole, has not been widely studied and was excluded from our studies.

## Conclusions

In conclusion, a systematic review failed to reach a consensus on whether stress-delta BNP or NT-proBNP reliably elevates in response to myocardial ischemia caused by a cardiac stress test. Our meta-analysis of the available data showed no statistical difference; however, the type of data reported in the literature added limitations to the meta-analysis model. Further studies with larger sample sizes and with longer post-stress time sampling will be necessary to better determine their utility in the assessment of potential ACS patients.
